# Choice perseverance underlies pursuing a hard-to-get target in an avatar choice task

**DOI:** 10.3389/fpsyg.2022.924578

**Published:** 2022-09-06

**Authors:** Michiyo Sugawara, Kentaro Katahira

**Affiliations:** ^1^Department of Cognitive and Psychological Sciences, Nagoya University, Nagoya, Japan; ^2^Japan Society for the Promotion of Science, Chiyoda-ku, Japan; ^3^Faculty of Letters, Arts and Sciences, Waseda University, Shinjuku-ku, Japan; ^4^National Institute of Advanced Industrial Science and Technology (AIST), Human Informatics and Interaction Research Institute, Tsukuba, Japan

**Keywords:** choice perseverance, pursuit behavior, avatar choice task, preference, reinforcement learning, hard-to-get

## Abstract

People sometimes persistently pursue hard-to-get targets. Why people pursue such targets is unclear. Here, we hypothesized that choice perseverance, which is the tendency to repeat the same choice independent of the obtained outcomes, leads individuals to repeatedly choose a hard-to-get target, which consequently increases their preference for the target. To investigate this hypothesis, we conducted an online experiment involving an avatar choice task in which the participants repeatedly selected one avatar, and the selected avatar expressed their valence reactions through facial expressions and voice. We defined “hard-to-get” and “easy-to-get” avatars by manipulating the outcome probability such that the hard-to-get avatars rarely provided a positive reaction when selected, while the easy-to-get avatars frequently did. We found that some participants repeatedly selected hard-to-get avatars (Pursuit group). Based on a simulation, we found that higher choice perseverance accounted for the pursuit of hard-to-get avatars and that the Pursuit group had significantly higher choice perseverance than the No-pursuit group. Model fitting to the choice data also supported that choice perseverance can account for the pursuit of hard-to-get avatars in the Pursuit group. Moreover, we found that although baseline attractiveness was comparable among all avatars used in the choice task, the attractiveness of the hard-to-get avatars was significantly increased only in the Pursuit group. Taken together, we conclude that people with high choice perseverance pursue hard-to-get targets, rendering such targets more attractive. The tolerance for negative outcomes might be an important factor for succeeding in our lives but sometimes triggers problematic behavior, such as stalking. The present findings may contribute to understanding the psychological mechanisms of passion and perseverance for one’s long-term goals, which are more general than the romantic context imitated in avatar choice.

## Introduction

People sometimes persistently pursue “hard-to-get” targets that do not easily provide the desired outcomes. For instance, scientists passionately pursue specific hypotheses that are difficult to prove for a long time (e.g., Singh, 1997). Even in the context of mate selection, people direct their unrequited passion to a person who does not respond positively and sometimes even pursue a specific person as a stalker (Meloy, 1999; Marazziti et al., 2015). However, it is poorly understood why people pursue hard-to-get targets.

Reinforcement learning has been widely used in a variety of areas to account for choice behavior in organisms (Doya, 2007). From a conventional reinforcement learning perspective, choice behaviors depend on previously obtained outcomes (Sutton and Barto, 1998; Daw and Tobler, 2014). According to this outcome-dependent process, an option that is never reinforced is rarely chosen. Thus, it is difficult to explain the pursuit of hard-to-get targets by reinforcement learning. From the computational perspective of reinforcement learning, previous studies have reported that asymmetric value updating leads to repetitive choices as follows: asymmetric value updating is able to facilitate the impact of positive outcomes and inhibit the impact of negative outcomes, subsequently leading individuals to repeat their previous choice (Katahira, 2018). However, hard-to-get targets rarely provide positive outcomes. On the other hand, humans and other animals have an inherent tendency to repeat their past choice independently of past outcomes (Lau and Glimcher, 2005; Schönberg et al., 2007; Akaishi et al., 2014; Alós-Ferrer et al., 2016; Erev and Haruvy, 2016). This tendency, called choice perseverance, is often incorporated into reinforcement learning models (Schönberg et al., 2007; Gershman et al., 2009; Katahira, 2015; Wilson and Collins, 2019; Sugawara and Katahira, 2021; Wang et al., 2022). We hypothesized that choice perseverance can account for the pursuit of hard-to-get targets more than asymmetric value updating.

If choice perseverance accounts for the pursuit of hard-to-get targets, are scientists and stalkers coasting on unattractive targets? Many studies have reported that choice *per se* increases the preference for a chosen target (Brehm, 1956; Ariely and Norton, 2008; Sharot et al., 2009; Izuma and Murayama, 2013; Cockburn et al., 2014; Schonberg et al., 2014; Koster et al., 2015; Nakao et al., 2016; Hornsby and Love, 2020). Through this choice-induced reevaluation, the chosen target becomes more preferred, which often leads an individual to choose the same option again. Therefore, we also hypothesized that if the target is continuously chosen due to choice perseverance, the target becomes recognized as more attractive.

This study aimed to investigate the above-mentioned hypotheses that choice perseverance accounts for the pursuit of hard-to-get targets and consequently increases the attractiveness of the pursued targets based on choice-induced reevaluation. We constructed an avatar choice task that mimicked partner selection in which the participants repeatedly select one avatar, and the selected avatar expresses the valence reactions through facial expressions and voice. We defined “hard-to-get” and “easy-to-get” avatars by manipulating the outcome probability such that hard-to-get avatars rarely provide a positive reaction when selected, while easy-to-get avatars frequently do. To control the baseline attractiveness of the avatars presented in the choice task, we selected avatars based on preference ratings that the participants provided before completing the choice task. Additionally, by manipulating the outcome probabilities (see section “Materials and methods” for further information), we established hard-to-get and easy-to-get avatars. The participants rated the attractiveness of the avatars again after the choice task, allowing us to examine whether attractiveness was altered by the choice task. All participants pursued the easy-to-get avatar, which frequently responded positively, whereas some participants pursued the hard-to-get avatar, which rarely responded positively. The simulation supported the hypothesis that the pursuit of the hard-to-get avatar was caused by higher choice perseverance. Subsequently, this hypothesis was empirically confirmed by fitting models to the experimental data.

## Materials and methods

### Participants

One hundred fifty participants were recruited *via* CrowdWorks.^1^ Due to the nature of our task, we only recruited participants who were at least 18 years old and were romantically interested in women. At the time this study was designed, there was no information regarding the effect size of the difference between participants who pursued and did not pursue the hard-to-get avatar. Therefore, the power analysis was conducted assuming that the difference between the two groups had a moderate effect size (0.25) (Cohen, 1988). The power analysis (*α* = 0.05 and *β* = 0.80) revealed that a minimum sample size of 64 participants per group was necessary. In addition, considering the possibility that the proportion of participants who pursued the hard-to-get avatar was relatively small, we decided to recruit 150 participants. The study was approved by the ethical research committee at Nagoya University and was carried out in accordance with relevant guidelines and regulations (NUPSY-200306-K-01).

### Online experimental procedures

Informed consent was obtained from all participants when they clicked “I Agree” after reading information regarding the aims and procedures of this study. After they completed the survey collecting basic demographic information, including gender and age, they downloaded the Inquisit player (Millisecond Software LLC, Seattle, WA, United States) and started a series of behavioral tasks (see the details below). To protect the participants’ privacy, all data were anonymized. If the participants completed the entire task and survey without interruption, we paid them 550 yen (approximately $5).

To exclude the effect of inappropriate choice behavior, the following exclusion criteria were applied: (i) the participants did not respond within the time limit (3,000 ms) in more than 30% of the total trials in the choice task, (ii) the participants’ choice reaction time was too short (less than 300 ms) in more than 30% of trials in the task, and (iii) the participants demonstrated a task-irrelevant choice pattern, such as alternating between left and right or always selecting only one side (even though each avatar randomly appeared on both sides). Only two participants were excluded from the subsequent analyses based on criterion (i). No participants met criterion (ii) or (iii). Thus, the data of 148 participants (129 males and 19 females; age: range = 18–65 years, mean ± SD = 38.07 ± 11.03) were analyzed.

### Behavioral task

#### Avatar evaluation task

**FIGURE 1:**
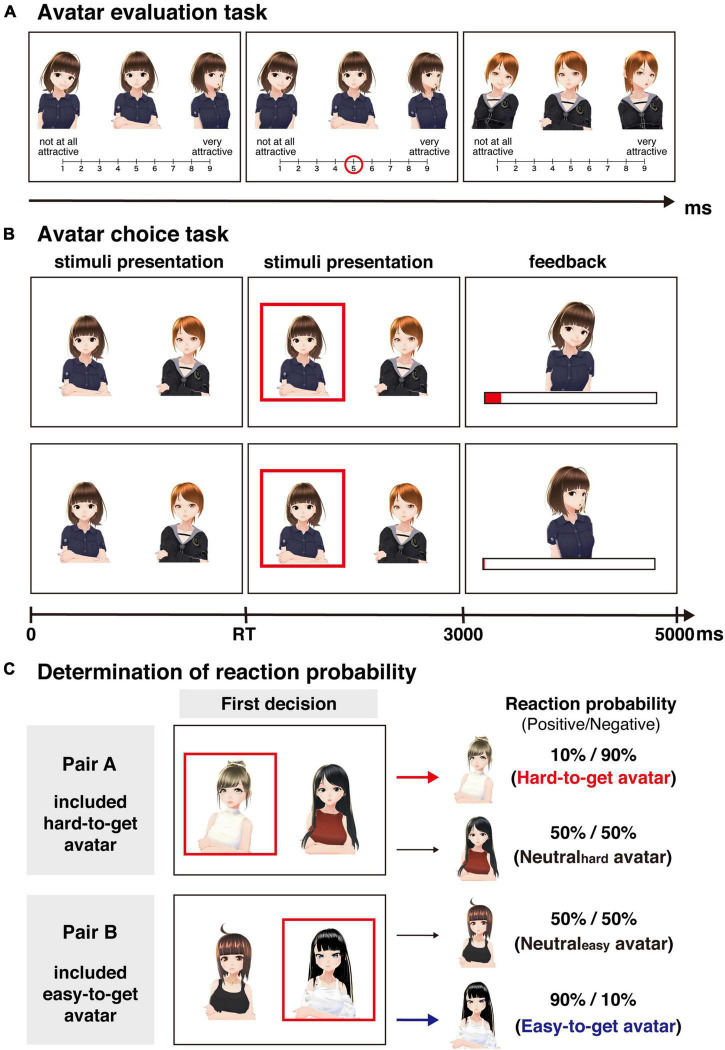
Behavioral tasks. **(A)** Avatar evaluation task. Participants were asked to rate the attractiveness of 48 avatars on a 9-point scale. **(B)** Avatar choice task. This task required participants to choose one of the two avatars displayed on the screen and to maximize the extent to which the avatar liked them, represented as a length of red bar. After they chose an avatar (RT is response time), the reaction from the chosen avatar was displayed. The upper line represents the flow of the task with a positive avatar reaction, while the lower line represents the flow of the task with a negative avatar reaction. Positive reactions increase likeability, while negative reactions do not alter likeability. **(C)** The outcome probability for each avatar was determined based on the first choice in the avatar choice task. In pair A, the initially chosen avatar was rarely associated with positive reactions in subsequent trials (positive/negative = 0.1/0.9; i.e., hard-to-get avatar). On the other hand, the initially chosen avatar in pair B was frequently associated with positive reactions in subsequent trials (positive/negative = 0.9/0.1; i.e., easy-to-get avatar). Avatar images reproduced with permission from Pixiv Inc.

In the online experiment, the participants performed the following two tasks: an avatar evaluation task and an avatar choice task. We created 48 avatars by using VRoid Studio (Pixiv Inc.^2^). First, the participants performed an avatar evaluation task in which they investigated the baseline attractiveness of 48 avatars. In this task (Figure 1A), the same avatar with different facial expressions (positive, neutral, and negative expressions) was displayed on the computer screen in a horizontal arrangement. For all avatars, the positive stimulus was a smiling facial expression, while the negative stimulus was a disappointed facial expression. The stimuli used in this task were identical across all participants. The participants were asked to rate the subjective attractiveness of the presented avatar on a 9-point scale (1: not at all attractive, 9: very attractive) by pushing numeric keys on their PCs. Such a point scale has also been used in previous studies investigating facial attractiveness (Shibata et al., 2016; Sakano et al., 2021). In this study, the initial evaluation task aimed to select avatars rated as more attractive than the middle point. Additionally, the second evaluation task aimed to assess the change in attractiveness compared to the initial evaluation task. We considered a 9-point scale a sufficient range to achieve these aims. The order of the presentation of the avatars was randomized across the participants. The full text of the instructions for the avatar evaluation task is shown in the Supplementary Material.

After the avatar evaluation task was completed, the participants performed the avatar choice task. To minimize the difference in the baseline attractiveness of the avatars used in the avatar choice task as much as possible, eight avatars were selected based on their attractiveness rating by the participants in the preceding avatar evaluation task. Avatars were selected following three steps.

• Step 1: Because it was important that the avatar used in this task was attractive to the participant (i.e., score of more than 6 points) and had the potential to become more attractive (i.e., did not score too high), avatars rated 6 or 7 points were selected.
• Step 2: If fewer than eight avatars were rated 6 or 7 points, among the avatars that rated less than 5 points, the avatar with the highest rating was selected.
• Step 3: If the total number of selected avatars was still less than eight, among the avatars rated more than 8 points, the avatar with the lowest rating selected. If there was more than one avatar with the highest rating among the avatars rated less than 5 points in Step 2 or with the lowest rating among the avatars rated more than 8 points in Step 3, an avatar was randomly selected from the avatars that fit the criteria. Then, Steps 2 and 3 were repeated in sequence until eight avatars were selected.

The participants again rated the attractiveness of the 48 avatars presented in the initial evaluation task after they completed the avatar choice task, allowing us to investigate whether the attractiveness of the avatars was altered after the choice task.

#### Avatar choice task

The avatar choice task consisted of two sessions. Based on the avatar evaluation task, we selected eight avatars that rated similar in attractiveness by each participant. Four pairs of avatars were randomly created from the eight selected avatars. Two pairs were used in the first session, and the remaining avatars were used in the second session. In each session, the participants completed 80 trials (40 trials/pair). The trials of both pairs were presented in a random order for each participant and session. That is, in each session, pairs A and B were presented in a mixed manner. In each trial (Figure 1B), the participants were required to choose one of two avatars with a neutral facial expression presented simultaneously on the screen for 3,000 ms. The presented position of the avatars was randomized across trials. After the participant selected an avatar, the selected avatar was highlighted with a red frame until 3,000 ms elapsed. In our previous study (Sugawara and Katahira, 2021), which investigated choice behaviors using non-meaning abstract images, the subjects were asked to choose one option for 2,000 ms. Some subjects did not complete their choice within this duration. Based on this experience, we extended the duration of choice to 3,000 ms in the present study. Then, the visual and auditory stimuli associated with the reaction of the selected avatar were presented for 2,000 ms. Specifically, the positive reaction was a smiling facial expression and a happy voice, while the negative reaction was a disappointed facial expression and a bored voice. When the reaction was displayed, a horizontal bar representing the accumulated liking from the avatar for the participant was presented below the avatar. At the beginning of the session, the width of this bar was zero for all avatars. Since the display size depended on the participant, the bar increased by 2% of the screen width when the avatar expressed a positive reaction, while the bar did not change when the avatar expressed a negative reaction. The participants were asked to maximize the extent to which the avatars liked them throughout the task. In the first trial of each avatar pair, the reaction of the selected avatar was always negative. In the following trials, the ratio of positive and negative reactions was determined by the first choice to minimize the influence of first impressions (Shteingart et al., 2013). For pairs A and B, the reaction probability (positive/negative) of the initially chosen avatar was set at 0.1/0.9 and 0.9/0.1, respectively. Based on this probability, we referred to the initially chosen avatar in pair A as the “hard-to-get” avatar and the initially chosen avatar in pair B as the “easy-to-get” avatar. For the unchosen avatars of both pairs (called “neutral_hard_” and “neutral_easy_”) in the first trial, the reaction probability was set at 0.5/0.5. These probabilities were fixed across the task. The participants were not informed in advance of the response probability of each avatar. Therefore, the participants were required to learn the response probability of each avatar (Figure 1C). The full text of the instructions for the avatar choice task is shown in the Supplementary Material.

### Behavioral analyses

We calculated the choice probability (hereafter CP) of avatars presented in the avatar choice task by dividing the number of choices by the number of trials (40 per avatar). Based on the CP of the hard-to-get avatar (CP_hard_), the participants were divided into the following two groups: the Pursuit group (CP_hard_ was more than 0.5) and the No-pursuit group (CP_hard_ was less than 0.5). Since there was no previous evidence regarding the pursuit of hard-to-get targets, the criteria for grouping were based on the chance level (i.e., 0.5). To confirm the difference in the CP of the hard-to-get and easy-to-get avatars between groups, two-way mixed-design analysis of variance (ANOVA) with group (Pursuit vs. No-pursuit) and avatar (hard-to-get vs. easy-to-get) was conducted.

To examine whether the baseline attractiveness differed among the avatars, including hard-to-get, easy-to-get, neutral_hard_, neutral_easy_, and unused avatars that were not presented in the choice task in the two groups, two-way mixed-design ANOVA with group (Pursuit vs. No-pursuit) and avatar (hard-to-get, easy-to-get, neutral_hard_, neutral_easy_, and unused) was performed. Additionally, to investigate whether attractiveness changed after the avatar choice task, we calculated the difference in the avatars’ attractiveness before and after the participants completed the avatar choice task. Then, two-way mixed-design ANOVA with group (Pursuit vs. No-pursuit) and avatar (hard-to-get, easy-to-get, neutral_hard_, neutral_easy_, and unused) was performed. To examine whether the attractiveness of each avatar changed, the degree of change in attractiveness was compared with zero using one-sample *t*-tests. The issue of multiple comparisons for one-sample *t*-tests was corrected with Bonferroni’s method. Moreover, to examine whether the choice *per se* increased the attractiveness of the chosen avatar, a general linear model analysis was performed. In this model, the change in attractiveness was a dependent variable. The changes in the attractiveness of the avatars used in the choice task were pooled across all participants. The number of choices, the number of positive reactions, and an interaction were independent variables.

All analyses were executed using R version 4.0.2 statistical software.^3^ Mendoza’s multisample sphericity test was used to check the validity of the sphericity assumption in all ANOVAs. To correct for violation of the sphericity assumption, Greenhouse–Geisser’s adjustment of the degrees of freedom was used in all ANOVAs when appropriate. *Post hoc* pairwise comparisons for significant effects were conducted based on Shaffer’s correction for multiple comparisons. The statistical threshold for significance was set at 0.05 for all behavioral analyses.

### Computational models

To investigate whether asymmetric value updating or choice perseveration better explains the pursuit of hard-to-get targets, we fit (i) asymmetric and (ii) perseveration models to the choice data. In addition, we previously demonstrated that a hybrid model including both asymmetric value updating and choice perseverance allows us to separately evaluate the contribution of these two cognitive processes to actual choice behavior (Sugawara and Katahira, 2021). Thus, we also fit (iii) a hybrid model. All models were modified based on a typical Q-learning model (called the “RL model”):


(1) $$\delta \left(t\right)=R\left(t\right)-Q_{i}\left(t\right),$$

(2) $$Q_{i}\left(t+1\right)=Q_{i}\left(t\right)+\alpha \delta \left(t\right).$$

Throughout this article, we usually consider cases with only two options (*i* = 1 or 2). The model assigns each option *i* an expected outcome *Q*_*i*_(*t*), where *t* is the index of the trial. The initial *Q*-values are set to zero [i.e., *Q*_1_(1) = *Q*_2_(1) = 0]. The model updates the *Q*-values depending on the outcome of the choice (i.e., the reaction of the chosen avatar). The actual outcome at trial *t* is denoted by *R*(*t*). We typically consider a binary outcome case whereby we set *R*(*t*) = 1 if a positive reaction is given and *R*(*t*) = 0 if a negative reaction is given. Learning rate *α* determines how much the model updates the action value depending on the reward prediction error, *δ*(*t*). Here, we denote the option that is chosen at trial *t* by *act*(*t*) (= 1 or 2). Based on the set of *Q*-values, the model assigns the probability of choosing option 1 using the softmax function:


(3) $$P\left(\mathrm{act}\left(t\right)=1\right)=\frac{1}{1+\exp\left(-\beta \left[Q_{1}\left(t\right)-Q_{2}\left(t\right)\right]\right)},$$

where *β* is called the inverse temperature parameter, which determines the sensitivity of the choice probabilities to differences in *Q*-values.

Based on the RL model, the asymmetric model assumes two independent learning rates:


(4) $$Q_{i}\left(t+1\right)=\left\{ \begin{array}{cc} Q_{i}\left(t\right)+\alpha^{+}\delta \left(t\right) & \;\text{if}\;\delta \left(t\right)\geq 0\\ Q_{i}\left(t\right)+\alpha^{-}\delta \left(t\right) & \;\text{if}\;\delta \left(t\right)<0, \end{array}\right.$$

where *α*^+^ adjusts the amplitude of value changes from one trial to the next when prediction errors are positive (when the actual reward *R*(*t*) is better than the expected outcome *Q*(*t*)); the changes with *α*^–^ are vice versa (Frank et al., 2007; Gershman, 2015; Palminteri and Lebreton, 2022).

The perseveration model is also based on the RL model and adds the computational process of choice history independent of the outcome-based learning process (Schönberg et al., 2007; Gershman et al., 2009; Akaishi et al., 2014):


(5) $$C_{i}\left(t+1\right)=C_{i}\left(t\right)+\tau \;\left(I\left(\mathrm{act}\left(t\right)=i\right)-C_{i}\left(t\right)\right).$$

Choice trace *C*_*i*_(*t*) is defined to introduce the effect of past choice into the CP. The initial values of *C*_*i*_(*t*) are set to zero [i.e., *C*_1_(1) = *C*_2_(1) = 0]. Indicator function *I*(⋅) takes a value of 1 if the statement is true and 0 if the statement is false. Decay rate *τ* is a free parameter that determines the number of preceding choices in the choice history influencing the current choice. When the choice is binary, the probability of choosing option 1 is implemented by the following:


(6) $$P(\mathrm{act}\left(t)=1\right)\;=\frac{1}{1+\exp\left(-\beta \left[Q_{1}\left(t\right)-Q_{2}\left(t\right)\right]-\varphi \left[C_{1}\left(t\right)-C_{2}\left(t\right)\right]\right)},$$

where the weight of choice history (*φ*) is a parameter that controls the tendency to repeat previous choices or avoid previously chosen options. A high positive value of this parameter indicates that the agent frequently repeats the previous choice.

Finally, the hybrid model has features of both asymmetric and perseveration models. This model incorporates not only asymmetric learning rates but also choice traces (equations 4–6). A previous study demonstrated that this hybrid model allows separate evaluation of asymmetric learning rates and choice perseverance (Katahira, 2018; Sugawara and Katahira, 2021).

### Simulation

To investigate what computational process contributes to the pursuit of the hard-to-get avatar, we simulated agents’ choices with the hybrid model. In particular, we systematically varied the free parameters of the hybrid model and evaluated CP_hard_ and CP_easy_ based on the simulated choice data. The task structure used in the simulation was identical to that in the online experiment.

The hybrid model had five parameters: learning rates for positive and negative reward prediction error (*α*^+^, *α*^–^), inverse temperature (*β*), decay rate (*τ*), and weight of choice history (*φ*). Because we were interested in the degree of asymmetric learning rates, the difference in learning rates (*α*_bias_ = *α*^+^ – *α*^–^) was calculated as the learning rate bias. In case 1, to examine the parameters related to the impact of past outcomes, the learning rate bias (−1 ≤ *α*_bias_ ≤1, interval = 0.1) and inverse temperature (0 ≤ *β* ≤ 10, interval = 1) were varied, but the decay rate (*τ* = 0.5) and the weight of choice history (*φ* = 1) were fixed. In case 2, to examine the parameters related to the impact of past choice, the decay rate (0 ≤ *τ* ≤ 1, interval = 0.1) and the weight of choice history (0 ≤ *φ* ≤ 10, interval = 1) were varied, but the learning rate bias (*α*_bias_ = 0) and inverse temperature (*β* = 2) were fixed. We hypothesized that the increased CP_hard_ would be accounted for by the higher choice perseverance, which was represented as the greater weight of choice history. Thus, we further examined the interaction between the weight of choice history and parameters related to the impact of past outcomes on the CP. In case 3, the learning rate bias (−1 ≤ *α*_bias_ ≤1, interval = 0.1) and the weight of choice history (0 ≤ *φ* ≤ 10, interval = 1) were varied, while the inverse temperature (*β* = 2) and the decay rate (*τ* = 0.5) were fixed. In case 4, the inverse temperature (0 ≤ *β* ≤ 10, interval = 1) and the weight of choice history (0 ≤ *φ* ≤ 10, interval = 1) were varied, while the learning rate bias (*α*_bias_ = 0) and the decay rate (*τ* = 0.5) was fixed. In the simulation, 100 virtual datasets were simulated for each parameter setting.

### Parameter estimation and model selection procedures

We fit the four models mentioned above (i.e., asymmetric, perseveration, and hybrid models) to the choice data derived from the avatar choice task. The standard RL model was also included as a benchmark for model fitting. Using the R function “solnp” in the Rsolnp package (Ghalanos and Theussl, 2015), we fit the parameters of each model with the maximum a posteriori estimation and calculated the log marginal likelihood for each model using the Laplace approximation (Daw, 2011). If all models have equal prior probability, because the marginal likelihood is proportional to the posterior probability of the model, the model resulting in the highest marginal likelihood is the most likely one given a dataset. Note that this study used the negative log marginal likelihood (i.e., lower values indicate a better fit). The prior distributions and constraints were set following previous studies (Palminteri et al., 2017; Sugawara and Katahira, 2021). All learning rates were constrained to the range of 0 ≤ *α* ≤ 1 with a *beta* (1.1, 1.1) prior distribution. The inverse temperature was constrained to the range of *β* ≥ 0 with a *gamma* (shape = 1.2, scale = 5.0) distribution. In the perseverance model, the decay rate was constrained to the range of 0 ≤ *τ* ≤ 1 with a *beta* (1, 1) distribution (i.e., a uniform distribution), and the choice trace weight was constrained to the range of −10 ≤ *φ* ≤ 10 with a *norm* (*μ* = 0, *σ*^2^ = 5) distribution.

For the model comparisons, two-way mixed-design ANOVA with group (Pursuit and No-pursuit) and model (RL, asymmetric, perseveration, and hybrid) was conducted to compare the log marginal likelihoods. Additionally, we compared the estimated model parameters. For the learning rates ($\alpha_{c}^{+}$, $\alpha_{c}^{+}$), two-way mixed-design ANOVA with group and valence was performed. To correct for the violation of the sphericity assumption, Greenhouse–Geisser’s adjustment of the degrees of freedom was used for the within-subject factor when appropriate. *Post hoc* pairwise comparisons were performed based on Shaffer’s correction for multiple comparisons. For the bias of learning rates, inverse temperature, decay rate, and weight of choice history, the group difference was evaluated using a two-sample *t*-test. All analyses were executed using R version 4.0.2 statistical software (see text footnote 3). The statistical threshold for significance was set at 0.05 for all comparisons of model fit and estimated model parameters.

## Results

The data of 148 participants (129 males and 19 females; age: range = 18–65 years, mean ± SD = 38.07 ± 11.03) were analyzed in the following steps. First, by calculating the CP in the avatar choice task, we investigated whether some participants pursued the hard-to-get avatars. Second, based on the subjective ratings of the attractiveness of the avatars, we tested the hypothesis that the pursuit of a specific avatar consequently increased the attractiveness of the pursued avatar. Third, to determine which cognitive processes (i.e., asymmetric value updating or choice perseverance) accounted for the pursuit of the hard-to-get avatar, we simulated hypothetical choice rates by varying the parameters of the hybrid model including the two cognitive processes (Sugawara and Katahira, 2021). Finally, to empirically confirm the prediction from the simulation, we fitted some variants of the reinforcement learning models to the actual choice data collected in the online experiment.

### Behavioral and subjective evaluation results

#### Choice probability in the avatar choice task

**FIGURE 2:**
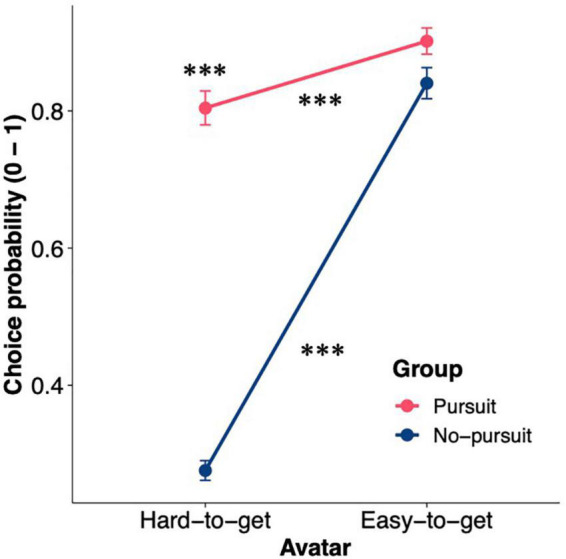
Choice probability for the hard-to-get and easy-to-get avatars in the two groups. The figure shows the choice probability for the hard-to-get and easy-to-get avatars in the two groups. The error bars represent the standard error of the mean. Asterisks above points denote whether the difference in choice probability between two groups for the avatar was significant, while asterisks above or below lines denote whether the difference in choice probability between two avatars was significant in each group: ^***^p < 0.001.

To characterize the participants who pursued the hard-to-get avatar despite frequent negative reactions, we focused on the choice probability of the hard-to-get avatar (CP_hard_) in the avatar choice task. For 68 of 148 participants, CP_hard_ was greater than 0.5 (i.e., they chose the hard-to-get avatar in more than half of the trials). Thus, we divided the participants into two different groups based on CP_hard_. The participants with a CP_hard_ value greater than 0.5 were assigned to the Pursuit group (*n* = 68; range = 0.50–1.00, mean ± SD = 0.80 ± 0.20), while the participants with a CP_hard_ value lower than 0.5 were assigned to the No-pursuit group (*n* = 80; range = 0.063–0.49, mean ± SD = 0.28 ± 0.12). The CP exhibited significant group differences between the hard-to-get and easy-to-get avatars [Figure 2; two-way mixed-design ANOVA; group × avatar interaction: *F*(1,146) = 144.29, *p* < 0.001, *η*^2^*_*G*_* = 0.31]. CP_hard_ in the Pursuit group was significantly higher than that in the No-pursuit group [simple main effect of group; *F*(1,146) = 385.66, *p* < 0.001, *η*^2^*_*G*_* = 0.73], confirming that the participants were grouped as intended. Meanwhile, CP_easy_ was comparable between groups [Pursuit group: range = 0.44–1.00, mean ± SD = 0.90 ± 0.17; No-pursuit group: range = 0.14–1.00, mean ± SD = 0.84 ± 0.20; simple main effect of group: *F*(1,146) = 3.87, *p* = 0.051, *η*^2^*_*G*_* = 0.026]. These results confirmed that the participants in the Pursuit group behaved differently only toward the hard-to-get avatar, whereas the participants in both groups repeatedly chose the easy-to-get avatar.

#### Attractiveness of avatars before and after the avatar choice task

If the baseline attractiveness of the initially chosen avatar was higher than that of the unchosen avatar, this difference in baseline attractiveness might have affected whether the participant pursued the hard-to-get avatar. However, our results indicated that the attractiveness of the avatars used in the choice task was not different between groups and that the paired avatars were rated at the same level of attractiveness in both groups. We compared the attractiveness rated before the choice task between avatars and groups. The baseline attractiveness for any type of avatar (i.e., hard-to-get, easy-to-get, neutral_hard_, neutral_easy,_ and unused avatars) was not significantly different between groups [Supplementary Figure 1; two-way mixed-design ANOVA; main effect of group: *F*(1,146) = 0.28, *p* = 0.60, *η*^2^*_*G*_* < 0.001; group × avatar interaction: *F*(1.4,204.05) = 0.32, *p* = 0.64, *η*^2^*_*G*_* = 0.001]. On the other hand, baseline attractiveness was significantly different among avatars [main effect of avatar: *F*(1.4,204.05) = 82.20, *p* < 0.001, *η*^2^*_*G*_* = 0.223]. The avatars used in the choice task were rated as more attractive than the unused avatars [*post hoc* pairwise comparisons; vs. hard-to-get avatar: *t*(146) = 9.15, *p* < 0.001, *d* = 0.76; vs. easy-to-get avatar: *t*(146) = 10.07, *p* < 0.001, *d* = 0.84; vs. neutral_hard_ avatar: *t*(146) = 9.56, *p* < 0.001, *d* = 0.80; vs. neutral_easy_ avatar: *t*(146) = 9.98, *p* < 0.001, *d* = 0.83; Shaffer corrected]. In addition, the avatars in pair B, including easy-to-get and neutral_easy_ avatars, had significantly higher attractiveness scores than the avatars in pair A, including hard-to-get and neutral_hard_ avatars [*post hoc* pairwise comparisons; easy-to-get vs. neutral_hard_ avatars: *t*(146) = 4.77, *p* < 0.001, *d* = −0.39; hard-to-get vs. easy-to-get avatars: *t*(146) = 3.80, *p* < 0.001, *d* = −0.32; neutral_hard_ vs. neutral_easy_ avatars: *t*(146) = 2.81, *p* < 0.05, *d* = −0.23; Shaffer corrected], with the exception of the comparison between hard-to-get and neutral_easy_ avatars [*post hoc* pairwise comparisons; *t*(146) = 0.46, *p* = 0.65, *d* = 0.04; Shaffer corrected]. However, the paired avatars had comparable attractiveness in the two groups [*post hoc* pairwise comparisons; hard-to-get vs. neutral_hard_ avatars: *t*(146) = 1.57, *p* = 0.24, *d* = −0.13; easy-to-get vs. neutral_easy_ avatars: *t*(146) = 2.12, *p* = 0.11, *d* = −0.17; Shaffer corrected].

**FIGURE 3:**
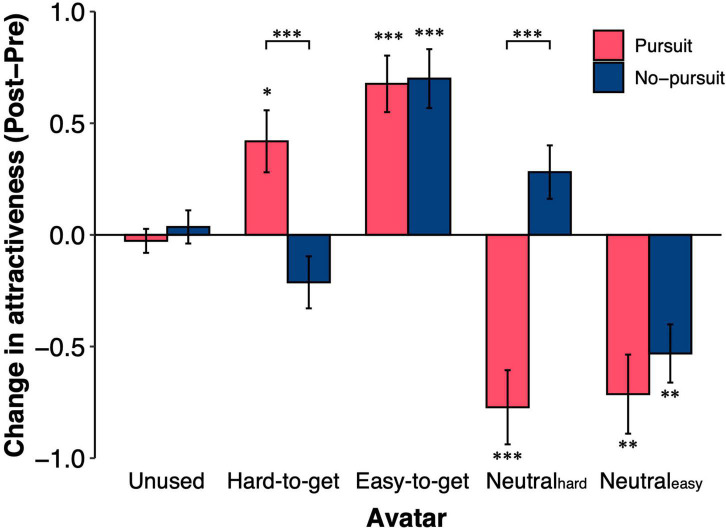
Changes in attractiveness ratings after the avatar choice task. The figure shows the changes in the attractiveness ratings of the five types of avatars in the two groups. Changes in attractiveness ratings were calculated by subtracting the score at the pre-choice rating from that at the post-choice rating. The unused avatars were not used in the avatar choice task (i.e., 40 avatars). The other types of avatars (i.e., hard-to-get, easy-to-get, neutral_hard_, and neutral_easy_) were used in the avatar choice task. The error bars represent the standard error of the mean. Asterisks denote whether the difference in attractiveness from before to after the avatar choice task was significant: ^***^p < 0.001, ^**^p < 0.01, and *p < 0.05.

We investigated how the attractiveness of the avatars changed through the choice task. The change in avatar attractiveness was calculated by subtracting the score before the choice task from the score after the choice task (Figure 3). The interaction between groups and the types of avatar was significant [group × avatar interaction: *F*(3.52,513.71) = 14.61, *p* < 0.001, *η*^2^*_*G*_* = 0.058]. The attractiveness of the unused avatars was not changed after the choice task in either group [one-sample *t*-test; Pursuit: *t*(67) = −0.50, *p* > 0.99, *d* = −0.06, No-pursuit: *t*(79) = 0.48, *p* > 0.99, *d* = 0.05; simple main effect of group: *F*(1,146) = 0.44, *p* = 0.51, *η*^2^*_*G*_* = 0.003]. The change in the attractiveness of the easy-to-get and neutral_easy_ avatars did not differ between groups [simple main effect of group; easy-to-get: *F*(1,146) = 0.016, *p* = 0.90, *η*^2^*_*G*_* < 0.001, neutral_easy_: *F*(1,146) = 0.71, *p* = 0.40, *η*^2^*_*G*_* = 0.004]. In both groups, the easy-to-get avatar was rated as more attractive [one-sample *t*-test, Pursuit: *t*(67) = 5.35, *p* < 0.001, *d* = 0.65; No-pursuit: *t*(79) = 5.31, *p* < 0.001, *d* = 0.59], while the neutral_easy_ avatar was rated as less attractive after the choice task [one-sample *t*-test, Pursuit: *t*(67) = −4.03, *p* < 0.01, *d* = −0.50; No-pursuit: *t*(79) = −4.08, *p* < 0.01, *d* = −0.46]. On the other hand, the attractiveness of the hard-to-get avatar increased in the Pursuit group [one-sample *t*-test, *t*(67) = 3.02, *p* = 0.036, *d* = 0.37], while it did not change in the No-pursuit group [one-sample *t*-test, *t*(79) = −1.82, *p* = 0.72, *d* = −0.20; simple main effect of group; *F*(1,146) = 12.33, *p* < 0.001, *η*^2^*_*G*_* = 0.078]. In contrast, the attractiveness of the neutral_hard_ avatar, which was paired with the hard-to-get avatar, decreased in the Pursuit group [one-sample *t*-test, *t*(67) = −4.65, *p* < 0.001, *d* = −0.56] but did not change in the No-pursuit group [one-sample *t*-test, *t*(79) = 2.35, *p* = 0.21, *d* = 0.26; simple main effect of group; *F*(1,146) = 27.55, *p* < 0.001, *η*^2^*_*G*_* = 0.159]. These results indicated that both hard-to-get and easy-to-get avatars were more attractive after the choice task in the Pursuit group, while only easy-to-get avatars were more attractive in the No-pursuit group.

The increased attractiveness of the hard-to-get and easy-to-get avatars in the Pursuit group raised the question of what events occurred in the choice task to increase the attractiveness of avatars. To answer this question, we conducted a general linear model analysis with the number of choices, the number of positive reactions, and an interaction with these numbers as independent variables and the changes in attractiveness as the dependent variable (see section “Materials and methods”). The number of choices had a significant effect only on the change in attractiveness observed after the choice task (*β* = 0.28, *p* < 0.001, *d* = 0.021). The main effect of the number of positive reactions (*β* = 0.063, *p* = 0.69, *d* = −0.018) and the interaction between the number of positive reactions and the number of choices were not significant (*β* = 0.005, *p* = 0.96, *d* < 0.001). Thus, the changes in attractiveness depended on the choice *per se* rather than reactions in the choice task.

### Simulation

We found that some participants (i.e., the Pursuit group) pursued the hard-to-get avatar despite receiving very few positive reactions. This behavioral phenomenon raised the question of which cognitive process led these participants to pursue the hard-to-get avatar. To answer this question, we used several variants of reinforcement learning models to determine what accounted for this choice behavior. As mentioned in the Introduction, asymmetric value updating (Lefebvre et al., 2017; Palminteri et al., 2017) and choice perseverance (Akaishi et al., 2014) can lead to repetitive choices of a previously selected option (Katahira, 2018). Thus, we conducted a simulation to investigate what parameters implemented in the hybrid model could account for the behavioral pattern shown in the Pursuit group. In particular, the hybrid model has five free parameters: learning rates for positive and negative reward prediction errors (*α*^+^ and *α*^–^), inverse temperature (*β*), decay rate of choice history (*τ*), and choice trace weight (*φ*) (see section “Materials and methods”). The degree of asymmetric value updating is denoted by the difference in the two learning rates (i.e., *α*_bias_ = *α*^+^ – *α*^–^). Thus, we simulated an agent’s choice behavior by manipulating these four parameters (*α*_bias_, *β, τ*, and *φ*) under the same task structure as the online experiment (see section “Materials and methods”).

**FIGURE 4:**
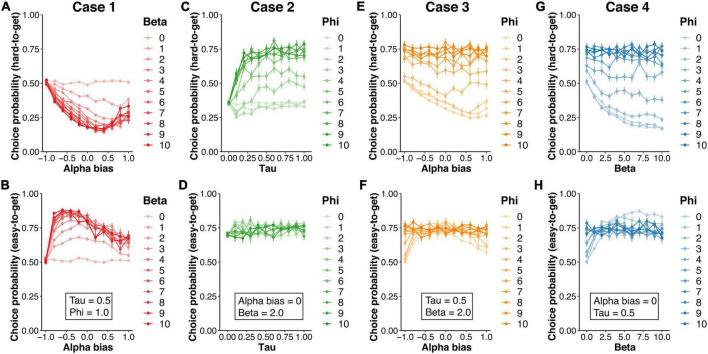
The results of the simulation in the hybrid model. The simulation of the agent’s choice behavior was generated by manipulating four parameters (*α*_bias_, *β, τ*, and *φ*) included in the hybrid model. The upper and lower rows show the choice probability for hard-to-get and easy-to-get avatars, respectively. In case 1, the bias of learning rates (*α*_bias_ = *α*^+^ – *α*^–^) and the inverse temperature (*β*) were varied, while the decay rate (*τ* = 0.5) and the weight of choice history (*φ* = 1.0) were fixed **(A,B)**. In case 2, *τ* and *φ* were varied, while *α*_bias_ (= 0) and *β* (= 2.0) were fixed **(C,D)**. In case 3, *α*_bias_ and *φ* were varied, while *φ* (= 1.0) and *β* (= 2.0) were fixed **(E,F)**. In case 4, *β* and *φ* were varied, while *α*_bias_ (= 0) and *τ* (= 0.5) were fixed **(G,H)**.

In case 1, *α*_bias_ and *β* were varied, while *τ* (= 0.5) and *φ* (= 1.0) were fixed. The asymmetric learning rates quadratically decreased CP_hard_ (Figure 4A) but quadratically increased CP_easy_ (Figure 4B). Moderate positivity bias (*α*_bias_ = 0.4) induced the smallest CP_hard_, while moderate negativity (*α*_bias_ = −0.6) bias induced the largest CP_easy_. The inverse temperature produced a linear decrease in CP_hard_ and a linear increase in CP_easy_. In any combination, CP_hard_ was less than 0.5, indicating that these parameters did not account for the behavioral pattern observed in the Pursuit group (CP_hard_ > 0.5).

In case 2, *τ* and *φ* were varied, while *α*_bias_ (= 0) and *β* (= 2.0) were fixed. For the hard-to-get avatar, CP_hard_ values in the condition with a moderate decay rate (*τ* > 0.2) and higher perseverance factor (*φ* > 6.0) reached over 0.7 (Figure 4C). Meanwhile, CP_easy_ did not depend on these parameters and was always over 0.7 (Figure 4D). Under the higher perseverance condition, the behavioral pattern was similar to that in the Pursuit group in the experiment.

To further examine whether the effect of perseverance trades off with the effect of the value-related parameters (i.e., *α*_bias_ and *β*), we covaried either *α*_bias_ (case 3) or *β* (case 4) with *φ*. In case 3, although CP_hard_ was modulated by the asymmetric learning rates (*α*_bias_) in the condition with lower perseverance (*φ* < 6.0), the condition with higher perseverance (*φ* > 6.0) showed higher CP_hard_ (Figure 4E) and CP_easy_ (Figure 4F). Likewise, in case 4, in the condition with higher perseverance (*φ* > 6.0), CP_hard_ (Figure 4G) and CP_easy_ (Figure 4H) were not affected by inverse temperature and showed higher probability (CP > 0.7). Therefore, these results suggested that higher perseverance was consistent with the behavior pattern shown in the Pursuit group.

### Model selection

To further investigate the mechanisms driving the pursuit of the hard-to-get avatar, we fit computational models to the choice data derived from the experiment. We used four variants of RL models to examine the benchmark of model fit: (1) a standard Q-learning model (hereafter, the RL model), (2) the asymmetric model, (3) the perseveration model, and (4) the hybrid model (see section “Materials and methods”). The results revealed that the perseveration model was the best for the Pursuit group, while the asymmetric model was the best for the No-pursuit group (Table 1). Mixed-design ANOVA showed a significant interaction between group and model [*F*(1.29,187.70) = 52.39, *p* < 0.001, *η*^2^*_*G*_* = 0.011]. There were no significant differences among the models in the No-pursuit group [simple main effect of model, *F*(1.05,83.28) = 0.12, *p* = 0.75, *η*^2^*_*G*_* < 0.001], but there were differences in the Pursuit group [simple main effect of model, *F*(1.57,105.27) = 91.00, *p* < 0.001, *η*^2^*_*G*_* = 0.036]. For the Pursuit group, there was no significant difference between the perseveration and hybrid models [*post hoc* comparison; *t*(67) = 0.47, *p* = 0.64, *d* = −0.06; Shaffer corrected]. However, the RL and asymmetric models, which did not include the choice history process, were much worse than the perseveration and hybrid models, which did include the choice history process [*post hoc* pairwise comparisons; RL vs. perseverance: *t*(67) = 11.83, *p* < 0.001, *d* = 1.44; RL vs. hybrid: *t*(67) = 10.42, *p* < 0.001, *d* = 1.26; RL vs. asymmetric: *t*(67) = 9.53, *p* < 0.001, *d* = 1.16; asymmetric vs. perseverance: *t*(67) = 9.52, *p* < 0.001, *d* = −1.15; asymmetric vs. hybrid: *t*(67) = 7.38, *p* < 0.001, *d* = −0.90].

**TABLE 1:** Models and model selection results.

Model	Learning rate (s)	Inverse temperature	Perseveration	No. of free parameters	Pursuit group LML (SD)	No-pursuit group LML (SD)
RL	*α* (*α*^+^ = *α*^–^)	*β*	–	2	−51.94 (27.52)	−53.83 (27.50)
Asymmetric	*α*^+^, *α*^–^	*β*	–	3	−47.09 (29.21)	−53.71 (27.47)
Perseveration	*α* (*α*^+^ = *α*^–^)	*β*	*τ, φ*	4	−37.89 (33.87)	−54.17 (27.23)
Hybrid	*α*^+^, *α*^–^	*β*	*τ, φ*	5	−38.23 (34.33)	−54.14 (27.29)

Furthermore, to examine whether the group difference in the underlying cognitive process was manifested in both pair A (including the hard-to-get avatar) and pair B (including the easy-to-get avatar), we separated the choice data of pairs A and B and then fit four models into the separated datasets (Table 2). The results showed a significant interaction between group and model in pair A [*F*(1.58,230.41) = 41.58, *p* < 0.001, *η*^2^*_*G*_* = 0.037] but not in pair B [*F*(1.51,220.14) = 1.19, *p* = 0.30, *η*^2^*_*G*_* < 0.001]. Although the simple main effect of model in pair A was significant in both groups [Pursuit: *F*(1.42,94.92) = 44.90, *p* < 0.001, *η*^2^*_*G*_* = 0.076; No-pursuit group: *F*(1.61,127.04) = 4.12, *p* = 0.026, *η*^2^*_*G*_* = 0.008], *post hoc* pairwise comparisons did not show any differences among models in the No-pursuit group (all *t* < 2.31, all *p* > 0.14, all *d* < 0.26). In contrast, there was a significant difference between all models in the Pursuit group [RL vs. asymmetric: *t*(67) = 11.30, *p* < 0.001, *d* = 1.37; RL vs. perseverance: *t*(67) = 9.09, *p* < 0.001, *d* = 1.10; RL vs. hybrid: *t*(67) = 6.80, *p* < 0.001, *d* = 0.83; asymmetric vs. perseverance: *t*(67) = 6.73, *p* < 0.001, *d* = −0.08; asymmetric vs. hybrid: *t*(67) = 4.42, *p* < 0.001, *d* = −0.54] with the exception of the comparison between the perseveration and hybrid models [*t*(67) = 1.32, *p* = 0.19, *d* = −0.16].

**TABLE 2:** Models and model selection results in each pair.

Condition	Model	Learning rate (s)	Inverse temperature	Perseveration	No. of free parameters	Pursuit group LML (SD)	No-pursuit group LML (SD)
Pair A includes	RL	*α* (*α*^+^ = *α*^–^)	*β*	–	2	−35.21 (12.23)	−34.37 (13.64)
hard-to-get	Asymmetric	*α*^+^, *α*^–^	*β*	–	3	−30.30 (15.06)	−34.19 (14.00)
avatar	Perseveration	*α* (*α*^+^ = *α*^–^)	*β*	*τ, φ*	4	−22.30 (21.06)	−37.14 (15.91)
	Hybrid	*α*^+^, *α*^–^	*β*	*τ, φ*	5	−23.51 (23.17)	−36.56 (15.16)
Pair B includes	RL	*α* (*α*^+^ = *α*^–^)	*β*	–	2	−16.57 (16.54)	−19.96 (15.95)
easy-to-get	Asymmetric	*α*^+^, *α*^–^	*β*	–	3	−15.74 (16.80)	−19.54 (16.36)
avatar	Perseveration	*α* (*α*^+^ = *α*^–^)	*β*	*τ, φ*	4	−17.33 (19.56)	−19.35 (16.48)
	Hybrid	*α*^+^, *α*^–^	*β*	*τ, φ*	5	−15.75 (19.61)	−19.50 (17.66)

These results indicate that the choice behaviors in the Pursuit group depended on the choice history, while the choice behaviors in the No-pursuit group did not show such a clear difference in history dependence. Furthermore, this group difference in the impact of choice history was observed only in the specific context involving avatars with relatively few positive reactions.

### Parameter estimation

To directly examine what computational process elicited the difference in choice behavior between the two groups, we compared the model parameters estimated from the hybrid model between groups. Although the hybrid model was not the best for the Pursuit and No-pursuit groups (Table 1), our previous study demonstrated that the hybrid model allows us to distinguish the effects of asymmetric value updating and choice perseverance (Sugawara and Katahira, 2021).

**FIGURE 5:**
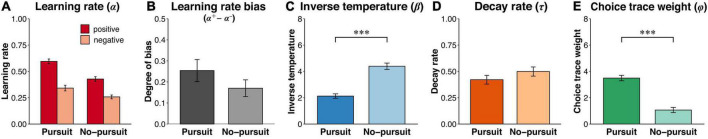
Estimated parameters with the hybrid model. The figure shows the estimated parameters by fitting the hybrid model to the choice data derived from the online experiment. **(A)** The learning rates for the positive and negative reward prediction errors (*α*^+^ and *α*^–^). **(B)** The learning rate bias calculated by subtracting the negative learning rate from the positive learning rate (*α*^+^ – *α*^–^), indicating the degree of asymmetric value updating. **(C)** The inverse temperature (*β*) representing the sensitivity to value differences in decision-making. **(D)** The decay rate (*τ*) indicating how far past choices are incorporated into the next choice. **(E)** The weight of choice history (*φ*) representing the sensitivity to differences in the choice history in decision-making. Error bars represent the standard error of the mean. Asterisks denote significant group differences: ^***^p < 0.001 and ^**^p < 0.01.

The Pursuit group had higher learning rates (*α*^+^, *α*^−^) than the No-pursuit group [Figure 5A; *F*(1,146) = 16.46, *p* < 0.001, *η*^2^*_*G*_* = 0.051]. Positive learning rates were higher than negative learning rates in both groups [*F*(1,146) = 42.85, *p* < 0.001, *η*^2^*_*G*_* = 0.133]. The interaction was not significant [*F*(1,146) = 1.67, *p* = 0.20, *η*^2^*_*G*_* = 0.006]. Furthermore, the difference between the positive learning rate minus the negative learning rate was calculated as the learning rate bias. There was no significant difference in the learning rate bias between groups [Figure 5B; *t*(146) = −1.29, *p* = 0.20, *d* = −0.21]. The inverse temperature (*β*) was significantly lower in the Pursuit group than in the No-pursuit group [Figure 5C; *t*(146) = 7.45, *p* < 0.001, *d* = 1.25]. While the decay rate (*τ*) was not significantly different between groups [Figure 5D; *t*(146) = 1.28, *p* = 0.20, *d* = 0.21], the choice trace weight (*φ*) was significantly higher in the Pursuit group than in the No-pursuit group [Figure 5E; *t*(146) = −8.48, *p* < 0.001, *d* = −1.40]. These results indicated that the Pursuit group placed greater weight on past choices than the No-pursuit group, while past outcomes had a greater influence on choice in the No-pursuit group than in the Pursuit group.

**FIGURE 6:**
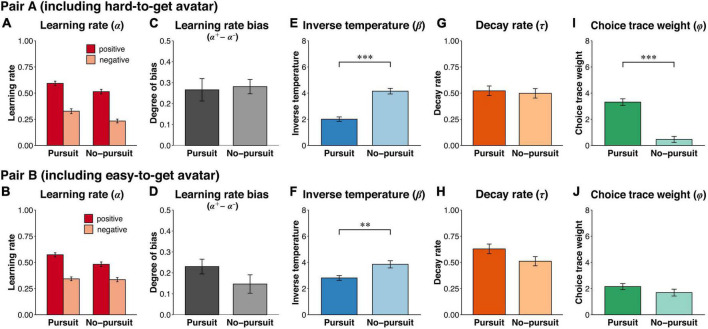
Estimated parameters with the hybrid model in each pair. The figure shows estimated parameters by fitting the hybrid model to the choice datasets separated by avatar pair. Upper and lower rows indicate estimated parameters for pair A (including the hard-to-get avatar) and pair B (including the easy-to-get avatar), respectively. **(A,B)** The learning rates for the positive and negative reward prediction errors (*α*^+^ and *α*^−^). **(C,D)** The learning rate bias calculated by subtracting the negative learning rate from the positive learning rate (*α*^+^−*α*^−^), indicating the degree of asymmetric value updating. **(E,F)** The inverse temperature (*β*) representing the sensitivity to value differences in decision-making. **(G,H)** The decay rate (*τ*) indicating how far past choices are incorporated into the next choice. **(I,J)** The weight of choice history (*φ*) representing the sensitivity to differences in the choice history in decision-making. Error bars represent the standard error of the mean. Asterisks denote significant group differences: ^***^p < 0.001 and ^**^p < 0.01.

To examine whether this group difference in choice perseverance was observed in a specific context, we compared the model parameters in each separate dataset. Regarding the learning rates with both pair A (Figure 6A) and pair B (Figure 6B), the main effect of valence was significant [pair A: *F*(1,146) = 78.36, *p* < 0.001, *η*^2^*_*G*_* = 0.222; pair B: *F*(1,146) = 42.88, *p* < 0.001, *η*^2^*_*G*_* = 0.128], whereas the interaction was not significant [pair A: *F*(1,146) = 0.06, *p* = 0.81, *η*^2^*_*G*_* < 0.001; pair B: *F*(1,146) = 2.10, *p* = 0.15, *η*^2^*_*G*_* = 0.007]. While the Pursuit group had a higher learning rate than the No-pursuit group with pair A [*F*(1,146) = 8.84, *p* < 0.01, *η*^2^*_*G*_* = 0.028], no significant difference was shown with pair B [*F*(1,146) = 2.93, *p* = 0.09, *η*^2^*_*G*_* = 0.010]. The learning rate bias was not significantly different with either pair [Figure 6C; pair A: *t*(146) = 0.24, *p* = 0.81, *d* = 0.40; Figure 6D; pair B: *t*(146) = −1.45, *p* = 0.15, *d* = −0.24]. The inverse temperature was significantly lower in the Pursuit group than in the No-pursuit group with both pairs [Figure 6E; pair A: *t*(146) = 7.64, *p* < 0.001, *d* = 1.28, Figure 6F; pair B: *t*(146) = 3.05, *p* < 0.01, *d* = 0.51]. The decay rate was not significantly different between groups [Figure 6G; pair A: *t*(146) = −0.37, *p* = 0.71, *d* = −0.06; Figure 6H; pair B: *t*(146) = −1.85, *p* = 0.07, *d* = −0.31] with both pairs. Importantly, while the choice trace weight was significantly higher in the Pursuit group than in the No-pursuit group with pair A [Figure 6I; *t*(146) = −8.41, *p* < 0.001, *d* = −1.39], there was no significant difference with pair B [Figure 6J; *t*(146) = −1.30, *p* = 0.20, *d* = −0.22]. The increased weighting for past choices shown in the Pursuit group was noticeable only in the context that included the hard-to-get avatar. The results suggested that an increased weight of past choices (i.e., higher choice perseverance) may lead to the pursuit of the hard-to-get avatar.

## Discussion

The present study investigated why people pursue hard-to-get targets. We hypothesized that choice perseverance, which is the tendency to repeat past choices, accounts for the pursuit of hard-to-get targets and consequently increases the attractiveness of the pursued targets. In the online experiment, the participants performed an avatar choice task to clarify the pursuit of hard-to-get targets. By manipulating outcome probabilities, we established the hard-to-get avatar as one that rarely had positive reactions and the easy-to-get avatar as one that frequently had positive reactions. For most participants, the easy-to-get avatars, which usually had positive reactions, were more frequently chosen than the paired avatars, which had positive and negative reactions at the same frequency. Nevertheless, some participants (i.e., the Pursuit group) frequently chose hard-to-get avatars that seldom had positive reactions and easy-to-get avatars. Thus, we confirmed that some people pursue hard-to-get targets. The participants also performed an avatar evaluation task to investigate increased attractiveness dependent on the choice *per se*. The attractiveness of the avatars after the choice task changed in accordance with the number of choices. Subsequently, following the choice task, the Pursuit group rated the hard-to-get avatar as more attractive, while the No-pursuit group rated this avatar as less attractive. Then, we used a computational modeling approach to reveal the cognitive process mediating the pursuit of the hard-to-get avatar. In a simulation, we demonstrated that a higher weight for choice history (i.e., choice perseverance) led to repetitive selection of not only the easy-to-get avatar but also the hard-to-get avatar. To confirm this finding in the empirical data, we fitted the hybrid model proposed in a previous study (Sugawara and Katahira, 2021) to the choice data derived from the online experiment. Consistent with the simulation results, the weight placed on choice history was significantly higher in the Pursuit group than in the No-pursuit group. According to these findings, we concluded that higher choice perseverance leads to repetitive choice of hard-to-get targets, consequently increasing the attractiveness of the selected target.

The primary finding of this study is that a part of participants pursued the hard-to-get avatar, which rarely provided positive outcomes. The pursuit of hard-to-get avatars in the Pursuit group was not explained by traditional reinforcement learning theory, which argues that the action probability is increased if the action is associated with positive outcomes (Thorndike, 1898; Sutton and Barto, 1998), even though the participants in the current experiment had to maximize the extent to which the avatar liked them. Another possible explanation is that the Pursuit group preferred the hard-to-get avatar over the alternative avatar because the baseline preference influenced their decision-making (Glimcher, 2009). However, in both groups, baseline attractiveness did not differ between the paired avatars used in the avatar choice task. Thus, differences in baseline attractiveness did not account for the pursuit of hard-to-get avatars. Choice perseverance reflects Thorndike’s law of exercise stating that producing an action makes it more likely to be selected on future occasions (Thorndike, 1898). Although the law of exercise captures the key feature of habits in which behavioral repetition automatizes behavior (Perez and Dickinson, 2020), habituation is due to reward-based learning mechanisms (Miller et al., 2019). Because the hard-to-get avatar seldom gave positive reactions, the pursuit of the hard-to-get avatar could not be accounted for by habituation. Unlike habituation, choice perseverance and the law of exercise are independent of choice outcomes. It is reasonable that the pursuit of the hard-to-get avatar is accounted for by choice perseverance.

Another important finding is that the increase in attractiveness depended on the number of choices rather than the number of positive reactions. This choice-dependent reevaluation has been reported (Brehm, 1956; Lieberman et al., 2001; Egan et al., 2007). Brehm (1956) reported that after a choice was made between two similarly valued options, the selected option was evaluated as better than the unchosen option. Choice-dependent reevaluation is usually accounted for by cognitive dissonance theory (Festinger, 1957). In this theory, when people choose one of two similarly desirable options, the conflict resulting from the desirability of the rejected option induces psychological distress. The reevaluation of the desirability of the chosen option occurs after choices are made to reduce such distress. One possibility is that the post-choice increases in avatar attractiveness found in this study might be accounted for by cognitive dissonance theory. However, previous studies observed choice-induced reevaluation even in amnesic patients who did not remember the option they chose (Lieberman et al., 2001), younger children, and capuchin monkeys (Egan et al., 2007). According to this evidence, it is reasonable that choice-induced reevaluation is mediated by a relatively simple and automatic process rather than complex cognitive reasoning. Sharot et al. (2009) showed that hedonic-related neural activity in the caudate nucleus in response to a selected option was enhanced after a decision was made in a free-choice task in which the participants freely choose between two options. This neuroimaging study suggests that imagination during the decision process activates hedonic-related brain regions and conveys pleasure expected from the simulated event. This choice-induced reevaluation modifies the hedonic response to the selected option. From the view of imagination-related pleasure, participants feel two types of pleasure in the avatar choice task used in this study: one induced by the imagination during the decision process and another induced by the obtained outcome. Participants with higher choice perseverance focus on the decision process rather than the obtained outcome. Thus, it is possible that their preferences are more strongly affected by the pleasure from imagination during the decision process, which consequently increases the attractiveness of the hard-to-get avatar.

Notably, some participants pursued hard-to-get targets, but others did not in the present study. Given that choice perseverance accounted for this group difference in pursuing hard-to-get targets, a critical question is how choice perseverance emerges. Although this important question remains unanswered, the present results could provide some insight into the context-dependency of choice perseverance. The computational modeling showed that the group difference in the weight of choice history (*φ*) was observed only in the choice context including hard-to-get avatars but not in the context including easy-to-get avatars. This finding suggests that the choice context modulated choice perseverance even among the participants who pursued hard-to-get avatars. The choice-dependent reevaluation mentioned above could be a potential source of choice perseverance. As discussed above, the choice-induced increase in the attractiveness of the chosen avatar might be mediated by value updating based on the pleasure derived from imagination during the decision process (Sharot et al., 2009). It is plausible that such imagination-based learning is emphasized by the lack of pleasure from the chosen outcomes (i.e., in the context including the hard-to-get target), leading to context-dependent choice perseverance. Future studies should investigate the fruitful hypothesis that imagination-based pleasure emphasizes choice perseverance, resulting in the pursuit of hard-to-get targets and the increased attractiveness of the pursued target.

Another important question is whether the pursuit of hard-to-get targets is specific to a social context or general in a broader decision-making paradigm. In this study, we adopted an avatar choice task to mimic the selection of romantic partners in real-life situations. It is possible that the specific effect of the social context, such as partner selection, leads to unexpected strategies and behaviors of a participant (e.g., perseverance to maintain self-image and not being directly upset by negative feedback). On the other hand, our previous study used an instrumental learning task with non-meaning simple symbols (Sugawara and Katahira, 2021). Even in a task with simple symbols, the degree of choice perseverance differed largely among individuals. According to our previous findings and the aforementioned evidence (Ghalanos and Theussl, 2015), some participants might pursue hard-to-get targets in a choice task with simple symbols. Furthermore, the avatar choice task used in this study was designed as a conventional two-armed bandit task. The participants must learn the outcome probability based on choice outcomes and maximize the outcomes obtained throughout the task. Thus, it was necessary to make the participants aware of the differences in the choice outcomes depending on the avatar’s response. To emphasize the difference between positive and negative outcomes, voice and facial expressions were also changed. If such a gamified nature of the avatar choice task contributes to pursuing hard-to-get targets, such pursuing behaviors might occur in a nonsocial choice task with gamified natures. Whether the degree of choice perseverance differs between social and nonsocial contexts even in the same participant remains largely unknown. To understand the effect of social contexts on cognitive processes underlying decision making, future studies should investigate whether choice perseverance differs between social and nonsocial contexts, or between with and without a gamified nature of the choice task.

We are able to evaluate the attractiveness of targets in various aspects such as physical, sexual, emotional, or aesthetic. Participants in this study were not instructed to rate attractiveness in terms of a specific aspect. It is possible that the aspects from which they rated attractiveness differed among participants. Nevertheless, the choice-dependent reevaluation of the avatar’s attractiveness was consistently found in both Pursuit and No-pursuit groups. We believe that individual differences in the aspect from which the attractiveness was rated did not have a significant effect on the present findings. Furthermore, in the present study, the apparent features such as hair and eyes were varied across avatars. Although the avatars used in the avatar choice task were selected based on the participant’s ratings preceding the choice task, it is possible that the differences in the apparent features between avatars might affect the choices if there was a bias in the apparent features. Indeed, numerous evidences show that avatars’ apparent features such as age, face shape, ethnicity, and eye/hair colors affect our perceptual responses (Messinger et al., 2008; Andrade et al., 2010; Turkay, 2012; Watson et al., 2012; Allison and Kendrick, 2013). The impact of these avatar’s apparent features on the choice behaviors remains an issue for future studies.

The present study has at least three limitations. The first issue is that statistical bias resulting from the free-choice paradigm might affect the choice-dependent attractiveness change observed in this study (Chen and Risen, 2010). Although the participants were repeatedly asked to choose one of the same paired avatars and received outcomes in the avatar choice task, the present experimental design is similar to a typical free-choice paradigm in which items are classified by preceding freely determined choices. The multiple regression analysis in the present study showed that the degree of attractiveness change depended on the number of choices of the avatar, which cannot be accounted for solely by statistical bias. Furthermore, a meta-analysis of the unbiased results (Izuma and Murayama, 2013) concluded that choice-induced preference change exists. However, to control for statistical bias resulting from a free-choice paradigm, forced-choice trials should be included in the avatar choice task. The second issue is that the amount of experienced positive outcome differed between the participants. As the avatar choice task was designed based on an instrumental learning framework in which the participants learn the outcome probability in a trial-and-error manner to maximize the obtained outcome, it is possible that the amount of experienced positive outcome depends on the chosen pattern and greatly varies among participants. To randomly choose two avatars at the beginning of each session, we introduced several tricks in the avatar choice task as follows: (1) avatars with a similar attractiveness were used, and (2) the initial reaction was always negative (see section “Materials and methods” for further information). Nevertheless, if the participant chose the hard-to-get avatar in all trials, the amount of experienced positive outcome from the hard-to-get avatar was more than that from another avatar that had never been chosen (Don et al., 2019; Don and Worthy, 2021). To solve this issue, the avatar choice task should be designed with an aligned number of reinforcements (Don and Worthy, 2021) or forced-choice trials should be incorporated (Niv et al., 2012). Future studies should confirm the present findings in more sophisticated task designs. The third issue is that most participants were male. Because of the abundance of avatar materials, we created avatars only with a female appearance. Thus, this study recruited subjects who were romantically interested in women. It remains unclear whether the present findings would be fully replicated even if the participants were female and the avatars were male in appearance. Future research needs to clarify whether the findings apply to female’s choice behavior.

The present study demonstrates that persons with higher choice perseverance pursued a target that rarely responded positively and consequently rated the selected target as more attractive *via* the choice-induced reevaluation mechanism. The tendency to pursue hard-to-get targets can be interpreted as tolerance for negative outcomes, contributing to grit (Duckworth et al., 2019). Tolerance for negative outcomes might be essential for success in our lives but sometimes triggers problematic behavior, such as stalking. The present findings shed light on the cognitive computational mechanisms underlying the pursuit of hard-to-get targets and may contribute to understanding the psychological substrates of grit constituted from passion and perseverance for one’s long-term goals, which are more general than the romantic context imitated in avatar choice (Duckworth et al., 2019).

## Data availability statement

The data supporting the findings of this study and the codes used for the simulation and model fits of the computational models are available in Figshare at https://figshare.com/articles/dataset/Pursuing_behavior_in_humans/13048478.

## Ethics statement

The studies involving human participants were reviewed and approved by the Ethical Research Committee at Nagoya University (NUPSY-200306-K-01). The patients/participants provided their written informed consent to participate in this study.

## Author contributions

MS conceived the study, performed the experiment, analyzed the data, and wrote the manuscript. MS and KK designed the experiment and interpreted the results. KK revised the manuscript. Both authors contributed to the article and approved the submitted version.

## Footnotes

1
https://crowdworks.jp/

2
https://vroid.com/en

3
http://cran.us.r-project.org
