# Supplementary material for: Choice perseverance underlies pursuing a hard-to-get target in an avatar choice task
Source: Front Psychol. 2022 Sep 6;13:924578. doi: 10.3389/fpsyg.2022.924578 (PMC9488557; doi:10.3389/fpsyg.2022.924578)
Supplement: Supplementary file 1 [file Data_Sheet_1.docx]

*Supporting Information*

# Supplementary Methods

## Instructions for the Avatar Evaluation Task (Translated into English)

In the avatar evaluation task, you will rate the attractiveness of avatars. After reviewing all expressions of the presented avatar, please press the numeric key corresponding to the degree of attractiveness of the avatar (1 = not at all attractive to 9 = very attractive). When you press the number key, the selected number will be highlighted in red. If you want to change the rating after pressing the number key, please press the "F" key. If you want to confirm the rating, please press the "J" key. Once the rating is confirmed, the avatar will change to the next one. Please repeat the evaluation in the same way.

**Caution**. If the data indicate that you performed this task in an inappropriate manner (e.g., just hitting keys repeatedly or not responding), we will not be able to give you the reward. Please be aware of this.

## Instructions for the Avatar Choice Task (Translated into English)

The avatar choice task consists of two parts. After the first 7-minute half, there is a 30-second break, followed by the second 7-minute half. In this task, some avatars rated in the avatar evaluation task appear in pairs. You should choose one of the presented pairs on the screen within 3 seconds. Press the “F” key on your keyboard if you choose the left avatar. Press the “J” key if you choose the right avatar. If it takes more than 3 seconds to choose an avatar, a warning message ("Please press within 3 seconds!") will appear. When you make a choice, the chosen avatar will be highlighted with a red frame. Once you have made a choice, you cannot change it. After making your choice, the feedback from the chosen avatar is displayed immediately. There are two types of feedback with a sound. If the avatar smiles at the time of feedback, the avatar’s liking for you increases. However, the avatar’s liking for you remains unchanged if the other type of feedback is displayed. The bar displayed below the avatar shows how much the avatar likes you. After the feedback disappears, the screen returns to the choice stage. You are asked to repeat the choice process throughout this task. Try your best to maximize the total amount of likability from avatars to you in the task. Even if the same pair appears, the position of each avatar may be changed to either the left or the right. Therefore, please look carefully before making your choice. In addition, the progress of the task does not change depending on the avatar’s position, feedback outcomes, or current likability level.

**Caution**. If the data indicate that you performed this task in an inappropriate manner (e.g., just hitting keys repeatedly or not responding), we will not be able to give you the reward. Please be aware of this.

# Supplementary Figures


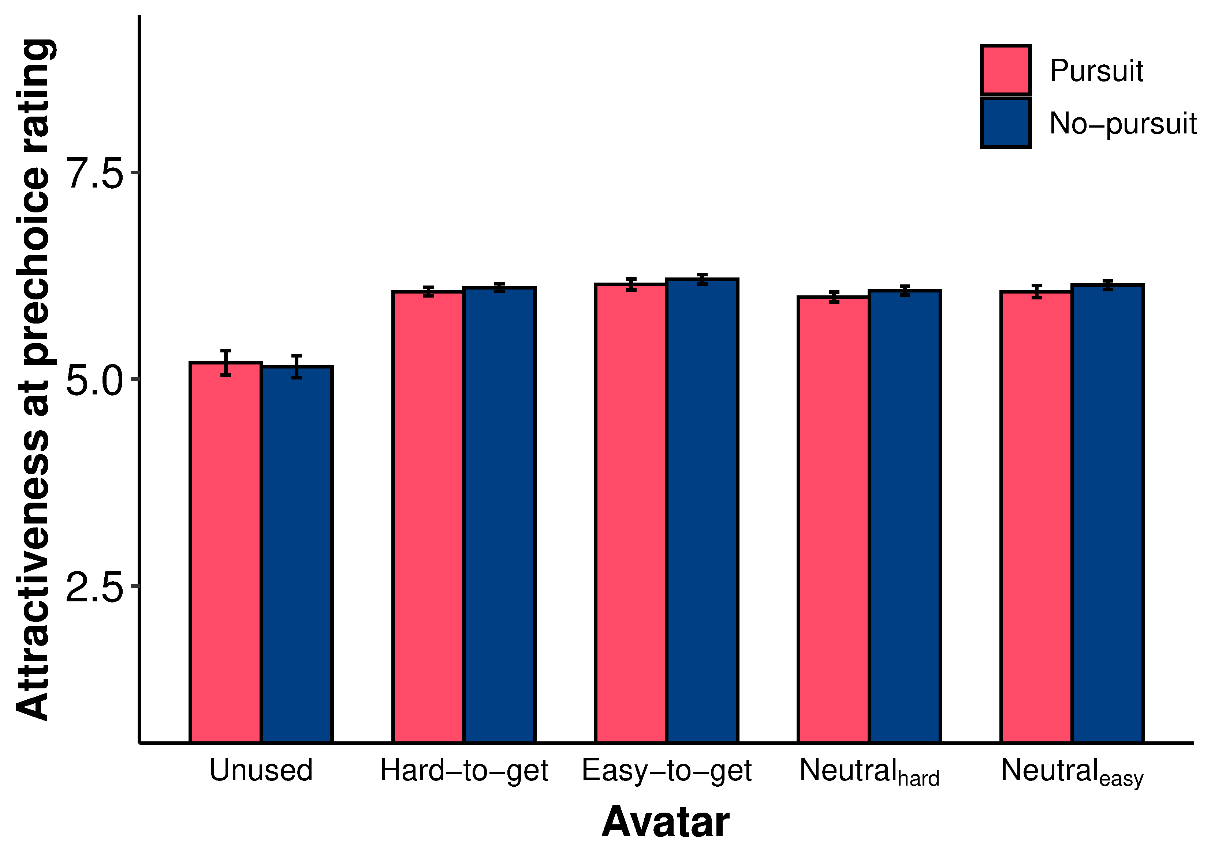


**Supplementary Figure 1.** This figure shows the attractiveness ratings of the five types of avatars according to the prechoice rating in the two groups. The unused avatars were not used in the avatar choice task (i.e., 40 avatars). The other types of avatars (i.e., hard-to-get, easy-to-get, neutral_hard_, and neutral_easy_) were used in the avatar choice task. The error bars represent the standard error of the mean.
